# Electro‐Co‐Polymerisation of Polypyrrole‐Polyaniline Composites in Ionic Liquids for Metal‐Free Hydrogen Evolution Electrodes

**DOI:** 10.1002/open.202400215

**Published:** 2024-09-24

**Authors:** Chhavi Sharma, Yuvraj Singh Negi, Kaushik Parida, Sara Dale

**Affiliations:** ^1^ Department of Physics University of Bath Bath UK; ^2^ Department of Polymer and Process Engineering Indian Institute of Technology Roorkee Saharanpur Campus 247001 India

**Keywords:** Copolymerization, Polymers, Hydrogen evolution, Electrodeposition, Ionic Liquids

## Abstract

Pure organic films consisting of polypyrrole, polyaniline and a composite of polypyrrole and polyaniline electrodeposited in the ionic liquid EMIM‐TFSI onto mesoporous carbon electrodes are tested for their hydrogen evolution reaction capabilities. The use of these intrinsically conducting polymers is seen as a way of stepping away from expensive and rare metallic catalysts. Co‐polymerisation of polypyrrole and polyaniline in a 1 : 10 ratio in EMIM‐TFSI was found to be doped with the TFSI^−^ anion and be much more active to the hydrogen evolution reaction when compared to pure polymers. Tafel analysis of the composite gave a value of 144 mV/dec indicating that the Volmer step is the rate limiting step. However, stability tests showed an improvement in the composite's overpoential performance for the hydrogen evolution reaction.

## Graphical abstract

Polypyrrole: polyaniline composites in a 1 : 10 ratio were studied for their hydrogen evolution ability and were found to improve after continued cycling in 0.5 M H_2_SO_4_ indicating protonation of the conducting polymers to a higher conducting state.

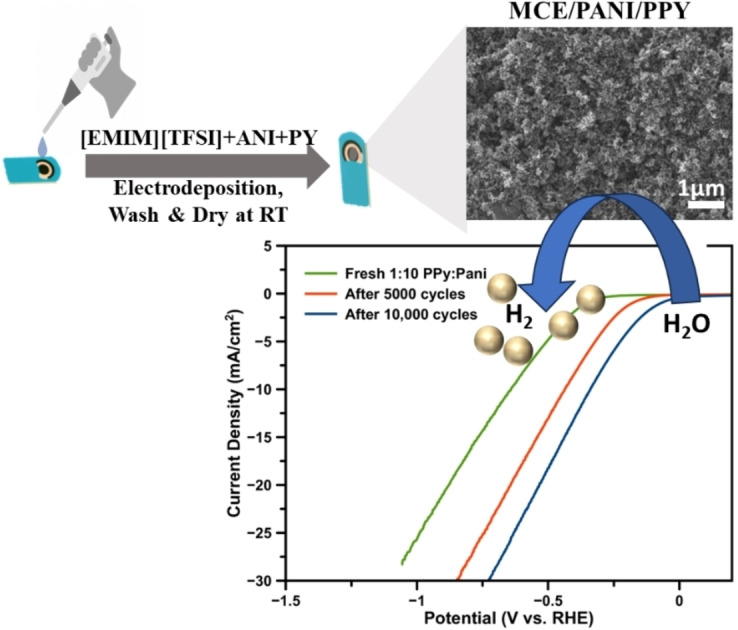

## Introduction

Intrinsically conducting polymers (ICPs) have the advantage of low cost, high availability with an ease of structural tunability and are widely seen as promising candidates for catalysing the hydrogen evolution reaction (HER).[^1^, ^2^, ^3^] ICPs have proven to be useful in many applications such as in flexible electronics,^[4]^ sensing,^[5]^ bioelectronics,[^6^, ^7^] supercapacitors,[^8^, ^9^, ^10^] and fuel cells.^[11]^ Polyaniline (Pani) and polypyrrole (PPy) are well‐studied ICPs which have shown good HER catalytic activity due to their conducting backbone and high electroactive surface area.[^12^, ^13^, ^14^] However, the morphology of the ICP is crucial to its catalytic properties and the ability to control this can be done in many ways including the electrochemical method used to deposit the ICP (cyclic voltammetry, chronoamperometry),^[15]^ concentration of starting monomer^[16]^ or the solvent the ICPs are deposited in.[^17^, ^18^]

Room temperature ionic liquids (RTIL) are widely seen as green solvents that are non‐volatile in nature with a high thermal stability and wide electrochemical window.[^19^, ^20^] They consist of bulky anions and cations and can be utilised as a solvent for the polymerisation process.[^21^, ^22^] ICPs electrodeposited in room temperature ionic liquids have shown high conductivity because of their ability to dope the ICPs.[^23^, ^24^] Previous studies into the electrodeposition of polyaniline and polypyrrole in ionic liquids have mainly focussed on the deposition of a single polymer.^[25]^ There are very few studies into the co‐deposition of two conducting polymers in an ionic liquid, however, Snook *et al*. found that co‐depositing polypyrrole with poly(3,4‐ethylenedioythiophene) (PEDOT) gave films with high ionic transport and a highly porous structure when compared to the single polymer films deposited in the same ionic liquid.^[26]^

Polyaniline and polyprrole composites made from aqueous solutions have been widely studied mainly for supercapacitor applications.[^27^, ^28^] Polyaniline and polypyrrole are both known to be highly stable in acidic conditions making them ideally suited to hydrogen evolution in acidic environments.[^29^, ^30^] Polyaniline in particular, can be converted from is emeraldine base form into a highly conducting state using acids.^[29]^ Polypyrrole is only marginally more conducting in acidic conditions but the incorporation of anions during polymerisation can make it highly conducting meanining that it is ideally suited to electropolymerisation in ionic liquids where there is an abundance of anions.^[30]^

In this study, we show the co‐polymerisation of polyaniline and polypyrrole electrodeposited in the ionic liquid 1‐ethyl‐3‐methylimidazolium bis(trifluoromethylsulfonyl)imide (EMIM‐TFSI) on mesoporous carbon screen printed electrodes. The use of ionic liquids as the electrodeposition medium for co‐polymerisation of polyaniline and polypyrrole will be explored for its role in the morphology of the resulting films as well as its doping properties. The highly porous films are characterised with Raman spectroscopy to confirm doping of the films and are tested for their hydrogen evolution ability with electrochemical methods.

## Experimental

### Materials

Mesoporous carbon screen printed electrodes (MC‐SPEs) were purchased from Metrohm (MC 110D). The monomers aniline (ACS reagent, >99.5 %) and pyrrole (Reagent grade, 98 %), as well as the ionic liquid 1‐ethyl‐3‐methylimidazolium bis(trifluoromethylsulfonyl)imide (EMIM‐TFSI, NMR grade, 97 %), sulfuric acid (95 %) and Pt/C (10 wt%) were all purchased from Sigma‐Aldrich and were used as received without further purification.

### Electrodeposition of Polypyrrole and Polyaniline Electrodes

Polyaniline, polypyrrole and 1 : 10 ratio polypyrrole: polyaniline composites were electrodeposited using a conventional three electrode setup where mesoporous carbon screen printed electrodes served as the working electrode and two platinum wires served as the pseudo‐reference and counter electrodes (Both were 1 mm dia., CH Instruments, UK). EMIM‐TFSI was first dried to remove any residual water by placing a small amount in a vial and placing in the oven antechamber attached to the glovebox (Vigor). The ionic liquid was heated to 100 °C for 30 minutes and then transferred to the glovebox which is kept under a nitrogen atmosphere with a water content to below 0.1 ppm and O_2_ to below 0.1 ppm. 0.5 M of the monomers of the ICPs were then dissolved in the dried ionic liquid and a small drop (2.5 μL) of this solution placed on the mesoporous carbon electrode to cover the entire electrode (4 mm dia.). Where the composite polymer was electrodeposited a solution containing 0.05 M pyrrole and 0.5 M aniline was made in the EMIM‐TFSI ionic liquid. The platinum counter electrode and reference electrode were then placed in the drop making sure not to touch each other. Electropolymerisation was carried out with the potentiodynamic method with a potential window of −0.4 V to +1 V at a scan rate of 50 mV/sec for 25 cycles using an Autolab PGSTAT 302 potentiostat. After electrodeposition had taken place, the working electrode was removed from the glovebox and washed with methanol/DI water and dried at room temperature. Pt/C electrodes were made by dispersing 10 wt.% Pt/C in methanol and drop casting this solution onto the mesoporous carbon electrode. Methanol was left to evaporate leaving the mesoporous carbon electrode with Pt/C nanoparticles on the surface.

### Characterisation of ICP Electrodes

ICP mesoporous carbon electrodes were characterised with Raman Spectroscopy (Renishaw inVia confocal Raman microscope) with a laser wavelength of 532 nm having a 1 % laser power and 10 accumulation cycles. Morphological analysis was done with FE‐SEM (Jeol JSM‐7900F FESEM) at an acceleration voltage of 5 kV. Structural analysis for as‐prepared electrodes was carried out with XRD (RIGAKU Oxford Diffraction SuperNova, Dual source) using Cu−Ka1 radiation at 1.5419 Å.

EIS measurements were recorded using an Autolab PGSTAT 302 with a frequency response analyser. The sinusoidal signal amplitude was ±10 mV and the frequency range was 100 kHz to 0.01 Hz. Circuit model fitting was carried out using Z view (II) software using a Randles circuit. Linear sweep voltammetry was recorded from 0 V to −1.5 V with a scan rate of 10 mV/s for the hydrogen evolution study. Tafel slopes were calulcated using the mesoporous carbon geometric area. Further electrode stability measurements were carried out using cyclic voltammetry (CV) at a scan rate of 50 mV/s for 10,000 cycles in an acidic medium (0.5 M H_2_SO_4_) in a three‐electrode setup with Ag/AgCl (sat. KCl) as the reference, carbon as the counter electrode and the modified mesoporous carbon SPE as the working electrode.

## Results and Discussion

### Electrodeposition of the ICP Electrodes using Cyclic Voltammetry

**Figure 1:**
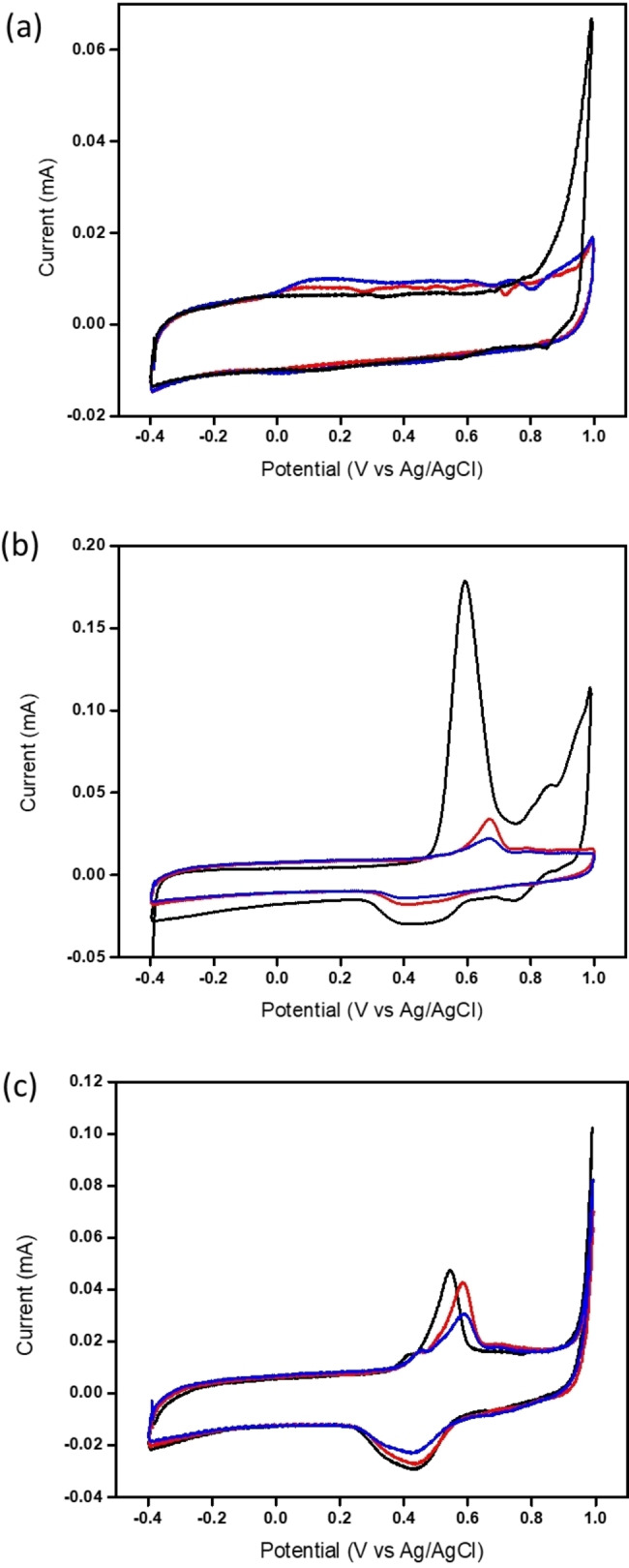
Cyclic voltammograms at 50 mV/s of the electrodeposition in EMIM‐TFSI on mesoporous screen printed electrodes of (a) 0.5 M polypyrrole, (b) 0.5 M polyaniline, (c) 0.5 M polyaniline and 0.05 M polypyrrole (1 : 10 ratio of polypyrrole: polyaniline). Black line is cycle 1, red line is cycle 10 and blue line is cycle 20.

Polypyrrole, polyaniline and composites of polypyrrole: polyaniline were synthesised using potentiodynamic cycling of the respective monomers in the ionic liquid EMIM‐TFSI. Figure 1(a) shows the cyclic voltammogram (CV) of the polymerisation of polypyrrole as being quite flat with only a small peak at 0.1 V (vs. Ag/AgCl) indicating the oxidation of the pyrrole monomer. Subsequent voltammograms show the peak to increase in current indicating the formation of a conducting polymeric film. This is consistent with the work from Pringle *et al*.,^[31]^ who found the peak for the electropolymerisation of polypyrrole in the ionic liquid EMIM‐TFSA to increase with successive cycles. They also found that the TFSA^−^ anion to intercalate in the polypyrrole during electropolymerisation thereby acting as a dopant for the film. It is well known that the TFSI^−^ anion dopes polypyrrole during electropolymerisation and optical microscopy of the pure polypyrrole films show a slight light brown colour on mesoporous carbon electrodes indicating the doped conducting state of polypyrrole (Supporting Information S1).^[32]^

The cyclic voltammetry (CV) of the electrodpolymerisation of polyaniline is shown in Figure 1(b). Here the first cycle shows a large peak at 0.6 V (vs. Ag/AgCl) for the oxidation of polyaniline from the leucoemeraldine state to the emeraldine salt, then a further peak at 0.82 V (vs. Ag/AgCl) for the oxidation of the emeraldine salt to the pernigraniline form of polyaniline.^[33]^ Cycles 2–20 show just a single peak at 0.67 V (vs. Ag/AgCl) which gradually decreases in current as the number of cycles increases. Due to the doping of polyaniline with EMIM‐TFSI, the polyaniline film does not reach its fully reduced form and therefore only 1 peak is seen for the oxidation of the emeraldine salt form of the polymer. The decrease in current also suggests that polyaniline is not as conductive as polypyrrole due to the porosity of the film not creating continuous conduction paths therefore acting to block the electrode rather than enhance it. Aniline polymerises under acidic conditions, however, in this case, no additional acid was added to the electrolyte for the polymerisation reaction. Imidazolium‐ring based ionic liquids are known to have an acidic proton in the C2 position of the ring^[34]^ and it is this acidic proton which contributes to the polymerisation reaction making this a more environmentally friendly way of synthesising polyaniline than traditional solvent/acid approaches. Optical microscopy of the resulting film shows a green colour suggesting that the polyaniline film is in the emeraldine salt form (Supporting information Figure S1). This will be discussed further in the Raman spectroscopy section.

**Figure 2:**
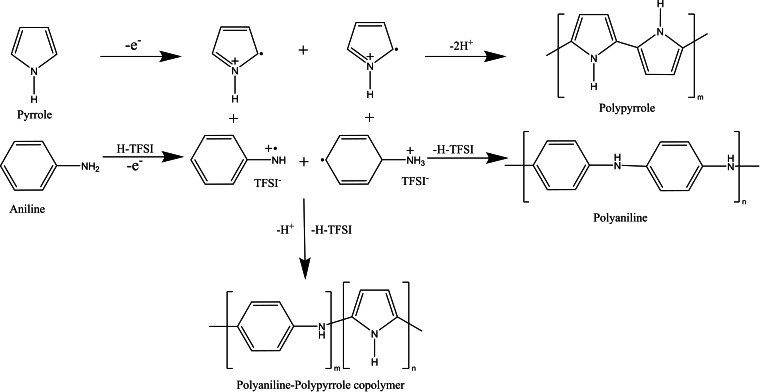
Reaction scheme for the polymerisation of polyaniline, polypyrrole and the copolymer polyaniline‐polypyrrole. Where m and n in the reaction scheme represent the number of repeated units in the polyme.

Polypyrrole electrodeposits at much lower potentials than polyaniline therefore an excess of aniline monomer was added to the pyrrole monomer in a 1 : 10 ratio of pyrrole: aniline. Figure 1(c), shows on the first cycle a peak at 0.52 V (vs. Ag/AgCl) with a small shoulder peak at 0.4 V (vs. Ag/AgCl). The shape of the CV is analogous to that of pure polyaniline but the first peak at 0.52 V for the conversion from the lercoemeraldine to the emeraldine salt form of polyaniline is greatly reduced on the first cycle. Subsequent cycles show the large polyaniline peak to rapidly stabilise at 0.6 V and the shoulder peak at 0.4 V. It is assumed that the shoulder peak is the polypyrrole formation as this peak does not decrease in current with number of cycles and shows the highly conducting polypyrrole is incorporated into the polyaniline film forming a copolymer. Figure 2 shows the mechanism for the copolymerisation reaction with the different structures of polypyrrole and polyaniline creating additional porosity within the polymeric film.

### Characterisation of the ICP Electrodes

**Figure 3:**
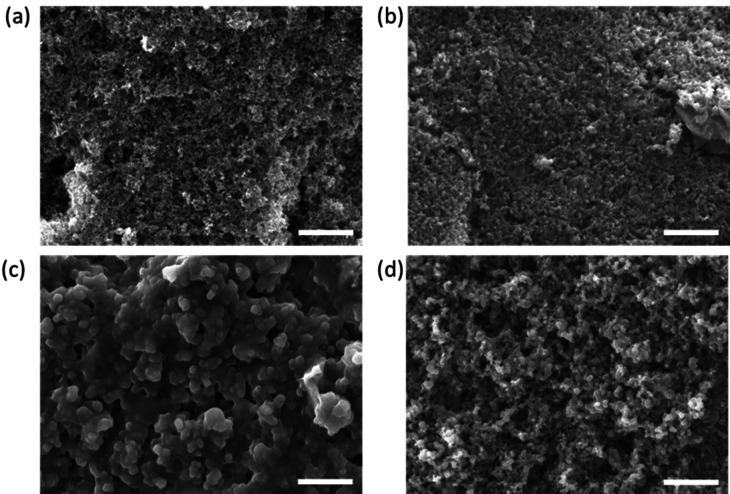
FE‐SEM images of the electrode where (a) bare mesoporous screen printed electrode, (b) 0.5 M polyaniline, (c) 0.5 M polypyrrole, (d) 0.5 M polyaniline and 0.05 M polypyrrole. The scale bar on the images is 1 μm.

The morphology of the electrode and conducting polymer deposits were characterised with FE‐SEM as shown in Figure 3. The bare mesoporous carbon electrode shows a highly porous network of carbon as expected which acts as a scaffold for the electrodeposition of conducting polymers (Figure 3(a)). Figure 3(b) shows the pure polyaniline deposit on the mesoporous carbon electrode which looks like a fine nanoparticle structure (ca. <5 nm) with high porosity. This highly porous structure means that the electrical interconnectivity between particles of polyaniline is reduced thereby making a less conducting film. This was seen with the CVs where the currents gradually decreased with increasing number of cycles thereby blocking the electrode surface. Pure polypyrrole deposits show a much thicker dense layer with a globular type structure (Figure 3(c)). On closer inspection the particles that make up the structure are approximately 100 nm in diameter meaning that many of the pores in the mesoporous carbon scaffold have been closed up reducing the film's ability for the hydrogen evolution reaction. However, the more dense structure allows for better electrical conductivity and explains the increase in currents seen in when cycling the potential during film formation. It can be seen from the FE‐SEM images in Figure 3(d) that there is a highly porous network of the composite polymer which is ideal for creating a high surface area for HER reactions. The reduction in the concentration of polypyrrole creates a finer more nanoparticle like structure (pore size of ca. 23 nm). This finer structure will increase the surface area further promoting sites for hydrogen production.

EDX analysis of polyaniline, polypyrrole and the composite polymer with a 1 : 10 ratio shows a large carbon peak which is expected for the conducting polymer structure as well as the mesoporous carbon electrode which was used as the scaffold to electrodeposit onto (Supporting information Figure S2). All three electrodes show fluorine, sulfur and nitrogen which are the constituents of the TFSI^−^ anion. This is consistent with work done previously showing the anion to dope the conducting polymer during electropolymerisation.^[35]^

**Figure 4:**
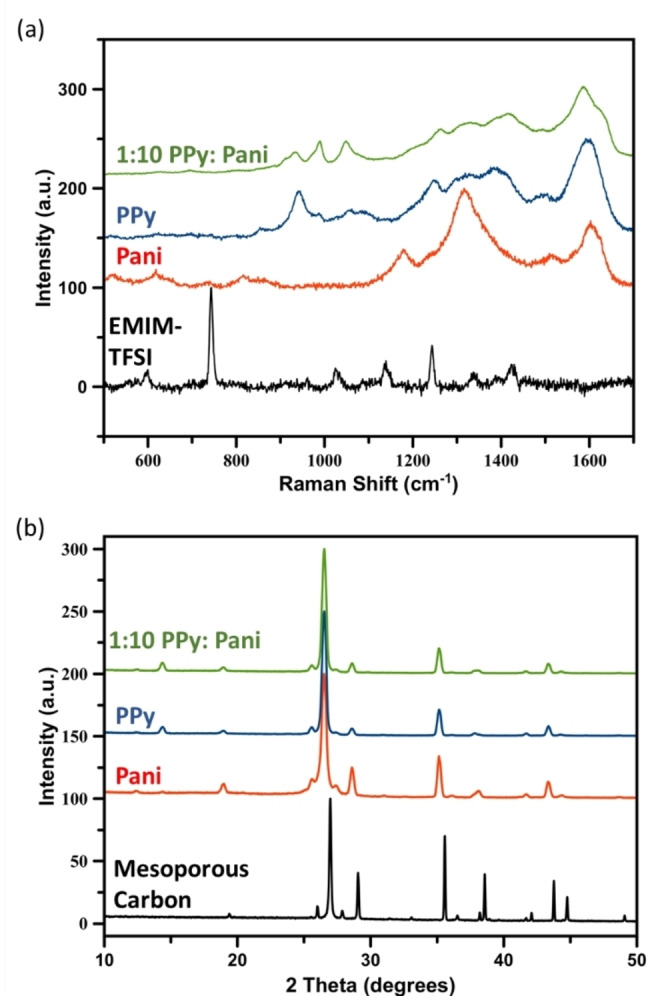
(a) Raman spectrum taken with a 532 nm laser of the ionic liquid EMIM‐TFSI, polyaniline, polypyrrole and 1 : 10 composite of polypyrrole: polyaniline films, (b) thin film XRD of the uncoated mesoporous carbon, polypyrrole, polyaniline and 1 : 10 polypyrrole : polyaniline composite films.

Figure 4(a) shows the Raman spectra for the ionic liquid EMIM‐TFSI, the individual polymers as well as the 1 : 10 composite film (Peak assignments can be found in Supporting Information S3). The spectrum for the EMIM‐TFSI shows mainly the peaks from the TFSI^−^ anion. There is a strong peak at 745 cm^−1^ which is the breathing mode of the TFSI^−^ anion with peaks at 1422 cm^−1^, 1395 cm^−1^ and 1339 cm^−1^ due to C−N stretching.[^36^, ^37^] Peaks at 1243 cm^−1^, 1139 cm^−1^ and 599 cm^−1^ are for the SO_2_ constituent of the TFSI anion which is consistent with previous Raman spectra of the EMIM‐TFSI ionic liquid.^[38]^

The Raman spectrum for pure polyaniline which has been electrodeposited from the monomer in the ionic liquid EMIM‐TFSI shows strong peaks at 1602 cm^−1^ and 1320 cm^−1^ due to the G and D band respectively of the mesoporous carbon substrate.^[39]^ These peaks dominate the spectrum, however, peaks belonging to the electrodeposited polyaniline in its emeraldine salt form are observed. For instance, peaks at 1238 cm^−1^ and 1174 cm^−1^ correspond to the C−N stretching and C−H benching of the emeraldine salt form of polyaniline.[^40^, ^41^] A small peak is seen at 742 cm^−1^ which belongs to the breathing mode of the TFSI^−^ anion.^[37]^ This indicates that the TFSI^−^ anion is still present in polyaniline films which is consistent with previous reports that this anion dopes electrodeposited polymer films.^[31]^

Figure 4(a) also shows the Raman spectrum for the pure polypyrrole films on mesoporous carbon electrodes. The peak at 1600 cm^−1^ and smaller peak at 1316 cm^−1^ are the G and D bands respectively of the mesoporous carbon electrode^[39]^ which again mask some of the peaks associated with polypyrrole. However, a peak at 1392 cm^−1^ is the ring stretching mode of polypyrrole and peaks at 979 cm^−1^ and 944 cm^−1^ are the symmetric and asymmetric ring deformation of pyrrole.[^42^, ^43^] The peak at ca. 750 cm^−1^ is absent in this spectrum indicating that the TFSI^−^ anion is not present, however, a peak at 1245 cm^−1^ is tentatively assigned to the SO_2_ antisymmetric stretching which is present in the TFSI^−^ anion.^[38]^ The presence of this peak suggests that the TFSI^−^ anion has broken up during electrodeposition but is still doping the polypyrrole film to a small extent.

Finally, the Raman spectrum for the 1 : 10 composite of polypyrrole: polyaniline was obtained and initially the peaks obtained look to be very similar to the polypyrrole spectrum. On closer inspection, there is a small shoulder peak at 1630 cm^−1^ which is attributed to the C−C stretching peak of polyaniline.^[40]^ Peaks at 985 cm^−1^ and 929 cm^−1^ are the polypyrrole ring deformation peaks and are much more pronounced than the polyaniline signal. This is to be expected as the electrodeposition of polypyrrole happens at much lower voltages to polyaniline. It is also interesting to note that there are a number of peaks associated with the EMIM‐TFSI indicating that there is a much higher amount of TFSI^−^ ions in these films. It is assumed that the higher porosity of the composite polymer films traps the ionic liquid into the pores.

XRD of the films is shown in Figure 4(b). The ordered mesoporous carbon substrate gives a number of diffraction peaks with the main peak being at 26° corresponding to the (002) diffraction of carbon and a smaller peak at 45° showing the (101) diffraction peak.^[44]^ Diffraction peaks at 38° and 44° indicate that the silver reference electrode which is printed on the screen printed electrode is also contributing to the diffraction spectrum and are attributed to the (111) and (200) diffraction of silver nanoparticles.^[45]^ XRD of the polymer films show largely the same diffraction peaks as the bare mesoporous carbon electrode, however, the large peak at 26° shows a slight broadening which is due to the amorphous nature of the polymer film.^[46]^ This is most pronounced in the polyaniline film but both the polypyrrole and composite polypyrrole‐polyaniline films show a slight broadening of the 26° peak when compared to the uncoated mesoporous carbon electrode showing that there is a polymer film on the electrode.

### HER Ability of the ICP Electrodes

**Figure 5:**
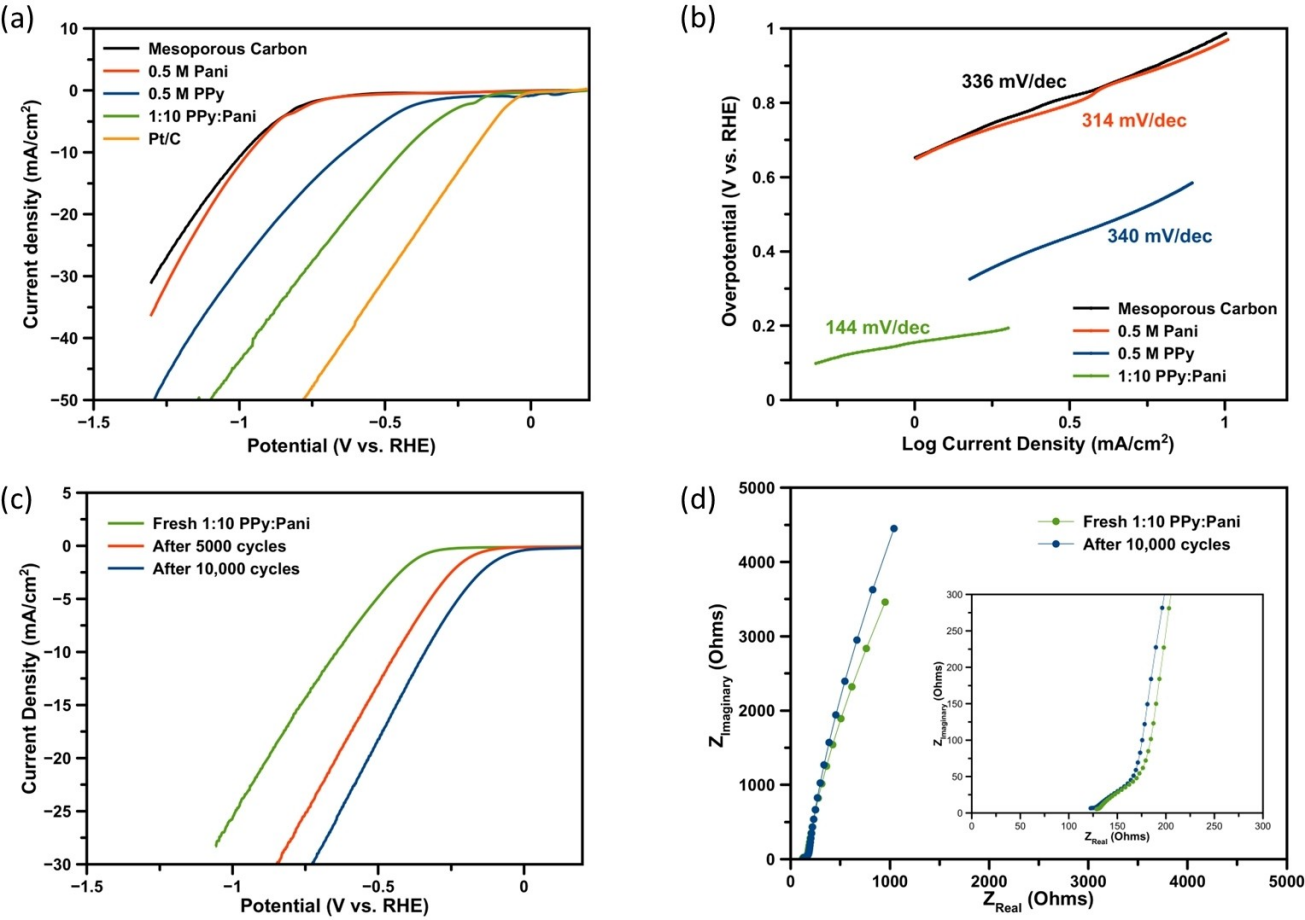
(a) linear sweep voltammetry of the polymer films along with 10 % Pt/C for comparison, (b) corresponding Tafel plots, (c) linear sweep voltammetry of the 1 : 10 polypyrrole: polyaniline film after 5,000 and 10,000 cycles, (d) electrochemical impedance spectroscopy of the 1 : 10 polypyrrole: polyaniline films before cycling and after 10,000 cycling for stability measurements with inset showing the high frequency end of the Nyquist plot.

The hydrogen evolution overpotential was obtained through running linear sweep voltammetry (LSV) in 0.5 M H_2_SO_4_ at a scan rate of 10 mVs^−1^. Figure 5(a) shows the LSVs for polyaniline, polypyrrole and the 1 : 10 polypyrrole: polyaniline composite as well as the bare mesoporous carbon electrode and a 10 wt.% Pt/C electrode. The mesoporous carbon electrode showed a high onset potential of −0.71 V (vs. RHE) for hydrogen evolution and the pure polyaniline also gave a large onset potential of −0.70 V (vs. RHE). The polyaniline electrode was a highly porous polymeric film and in the cyclic voltammetry in Figure 1(b), the currents drastically decreased upon further deposition indicating a partial blocking of the electrode surface. The high porosity of the film decreases the films electrical conductivity and therefore its ability to perform hydrogen evolution. The pure polypyrrole film showed a much lower onset potential of −0.45 V (vs. RHE) showing the good electrical connectivity of the film. The 1 : 10 polypyrrole: polyaniline composite film showed a much lower onset potential of −0.31 V (vs. RHE) meaning that the composite film has higher hydrogen evolution activity when compared to the pure polymers.

Tafel analysis of the polymer films gave very high Tafel slopes for the pure polymers. The polyaniline and polypyrrole had Tafel slopes which were comparable to the bare mesoporous carbon electrode, 314 mV/dec, 340 mV/dec and 336 mV/dec respectively, indicating that any increase in surface area by the electrodeposition of the polymer was outweighed by the decrease in electrical conductivity of the film. However, the composite polymer showed a much lower Tafel slope of 144 mV/dec as it has the optimum balance between high surface area and electrical conductivity. This Tafel slope is comparable to previous studies by Aydin *et al*. who found a Tafel slope of 124 mV/dec for composite films of polyaniline and polypyrrole for hydrogen evolution in 0.5 M H_2_SO_4_.^[47]^ A 1 : 1 ratio and a 1 : 20 ratio of polypyrrole: polyaniline was also tested and were found to have high Tafel slopes of 190 mV/dec and 231 mV/dec respectively showing that the 1 : 10 ratio has the optimum performance for hydrogen evolution (Supporting information Figure S4). The difference in Tafel slopes between the 1 : 1 ratio and 1 : 10 ratio of the composites is attributed to the globular structure of the polypyrrole reducing the porosity and therefore surface area of the electrode for hydrogen evolution. The 1 : 10 ratio has a reduced polypyrrole content and therefore a more open structure allowing for a greater surface area for hydrogen evolution. The 1 : 20 ratio has a much higher Tafel slope which is more akin to the pure polyaniline because the polypyrrole is in such low concentration. A high Tafel slope indicates that the Volmer step is the rate limiting step in the hydrogen evolution reaction and could be due to the slow electron transfer kinetics of the composite polymer film.^[48]^

Upon potentiodynamic cycling of the 1 : 10 polypyrrole: polyaniline electrode in 0.5 M H_2_SO_4_, it was found that the onset potential for hydrogen evolution reduced (Figure 5(c)). After 5,000 cycles, the onset potential had reduced to −0.25 V (vs. RHE) and after 10,000 cycles, this had reduced further to −0.17 V (vs. RHE). This high electrochemical stability of conducting polymer films fabricated in ionic liquids was also shown by Lu *et al*. who saw greatly enhanced lifetimes of polyaniline and polypyrrole films in ionic liquids.^[49]^ Electrochemical impedance spectroscopy of films before and after cycling are shown in Figure 5(d) and indicate that the change in the composite films is largely at the low frequency part of the Nyquist plot. Films measured after cycling show behaviour which has a higher Warburg diffusional impedance suggesting that the pores have closed (Supporting information Figure S5). FE‐SEM images of the composite film after cycling show the morphology of the film to remain largely unchanged compared to the fresh film indicating that any changes in electronic ability is down to doping rather than physical film changes (Supporting information Figure S5). Continued cycling in acidic media protonates the polyaniline film and could be expelling the TFSI^−^ anion from the composite film. The polyaniline in the composite film could be converting more of the film into the highly conductive emeraldine salt form making the film more conductive for hydrogen evolution.^[50]^ Tafel analysis of the composite films after stability tests show a slight increase in slopes measured but still much lower than individual polymers (Supporting information Figure S5). This could be due to the decrease in the diffusion, seen in the impedance spectroscopy measured, hindering the ability for hydrogen to adsorb on the surface of the polymer film.

## Conclusions

Highly porous films of polyaniline, polypyrrole and 1 : 10 polypyrrole: polyaniline were electrodeposited onto mesoporous carbon electrodes. The use of ionic liquids as a medium to electrodeposit from has shown to be advantageous for doping of these ICPs with the TFSI^−^ anion shown to incorporate into the films during electrodeposition. A 1 : 10 ratio of polypyrrole: polyaniline was found to be more active towards hydrogen evolution than the pure polymers with a Tafel slope of 144 mV/dec for the composite. Composite films were also shown to be highly stable and the onset potential for hydrogen evolution reduced from −0.31 V to −0.17 V (vs. RHE) upon continuous cycling. This could be due to the increased protonation of the polyaniline in the composite film making the film more conductive but more experiments are needed to confirm this.

## Conflict of Interests

The authors declare no conflict of interest.

1

## Supporting information

As a service to our authors and readers, this journal provides supporting information supplied by the authors. Such materials are peer reviewed and may be re‐organized for online delivery, but are not copy‐edited or typeset. Technical support issues arising from supporting information (other than missing files) should be addressed to the authors.

Supporting Information

## Data Availability

The data that support the findings of this study are available from the corresponding author upon reasonable request.
