# Supplementary material for: Electro‐Co‐Polymerisation of Polypyrrole‐Polyaniline Composites in Ionic Liquids for Metal‐Free Hydrogen Evolution Electrodes
Source: ChemistryOpen. 2024 Sep 24;13(12):e202400215. doi: 10.1002/open.202400215 (PMC11625919; doi:10.1002/open.202400215)
Supplement: Supplementary file 1 — Supporting Information [file OPEN-13-e202400215-s001.pdf]

ChemistryOpen

Supporting Information

Electro-Co-Polymerisation of Polypyrrole-Polyaniline Composites in Ionic Liquids for Metal-Free Hydrogen Evolution Electrodes

Chhavi Sharma, Yuvraj Singh Negi, Kaushik Parida, and Sara Dale*

Supporting Information

Electro-co-polymerisation of Polypyrrole-Polyaniline Composites in Ionic Liquids for Metal-Free Hydrogen Evolution Electrodes

Chhavi Sharma [a,b], Yuvraj Singh Negi[b], Kaushik Parida[b], Sara Dale[a*]

Figure S1.

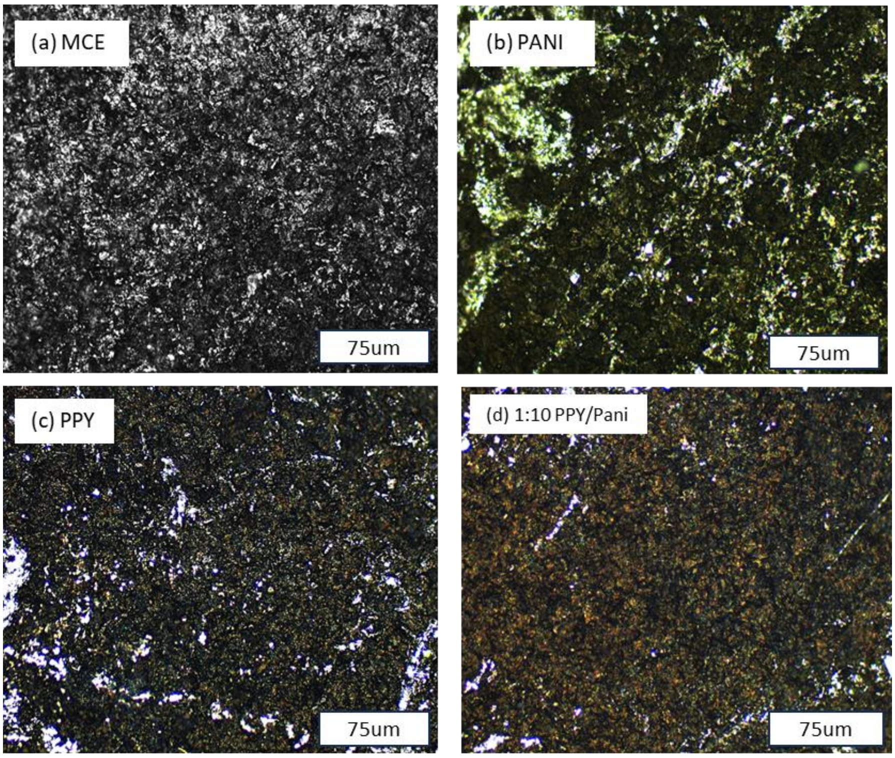

Figure S1. Optical microscopy images of (a) the bare mesoporous screen printed electrode, (b) 0.5 M polyaniline film, (c) 0.5 M polypyrrole film and (d) 1:10 polypyrrole:polyaniline film.

Figure S2.

(a)

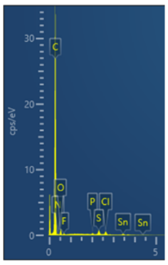

Element	Line Type	Atomic %
C	K series	80.9
N	K series	12.78
O	K series	4.60
F	K series	0.66
S	K series	0.43
Cl	K series	0.35

(b)

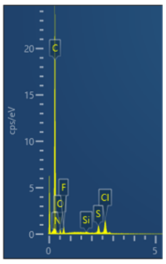

Element	Line Type	Atomic %
C	K series	88.78
N	K series	4.54
O	K series	2.76
F	K series	1.99
S	K series	0.64
Cl	K series	1.23

(c)

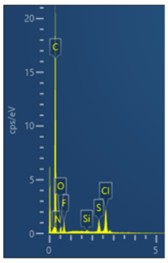

Element	Line Type	Atomic %
C	K series	86.28
N	K series	4.34
O	K series	3.89
F	K series	2.80
S	K series	0.86
Cl	K series	1.77

(d)

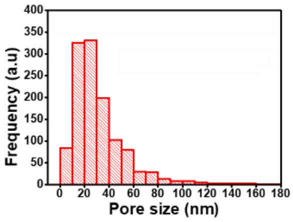

Figure S2. EDX analysis of (a) 0.5 M polyaniline, (b) 0.05 M polypyrrole and (c) 1:10 polypyrrole:polyaniline composite film (d) pore size histogram of the 1:10 polypyrrole: polyaniline film.

Figure S3.

Material	Wavenumber /cm^{-1}	Raman Mode	Ref
EMIM-TFSI	1422, 1395, 1339	C-N Stretching	[1,2]
	1243	SO_2 antisymmetric stretching	[3]
	1139	SO_2 symmetric stretching	[3]
	1027	C-N ring bend	[1,2]
	750	TFSI anion breathing	[1,2]
	599	SO_2 antisymmetric bending	[3]
Polyaniline (Emeraldine Salt form)	1621, 1614	C-C stretching	[5]
	1602	G band in mesoporous carbon	[4]
	1574	C=C stretching	[5]
	1505	C-C stretching	[5]
	1316	D band in mesoporous carbon	[4]
	1238	C-N stretching in ES form	[5,6]
	1174	C-H stretching in ES form	[5,6]
	856	B deformation	[5]
	809	C-N-C bending	[5]
	742	$TFSI^-$ anion breathing	[1,2]
	644	Ring deformation	[5]
	526	Amine in plane deformation	[5]
Polypyrrole	1601	G band in mesoporous carbon	[4]
	1503	C-C stretching	[7]
	1392	Ring stretching mode of PPy	[7]
	1316	D band in mesoporous carbon	[4]
	1245	SO_2 antisymmetric stretching of $TFSI^-$	[2]
	1094, 1062	C-H in plane deformation	[7]
	979	Pyrrole ring deformation symmetric	[8]
	944	Pyrrole ring deformation asymmetric	[8]
1:10 Polypyrrole: Polyaniline composite	1630	C-C stretching in Pani	[5]
	1591	G band in mesoporous carbon	[4]
	1409, 1394, 1337	C-N stretching in $TFSI^-$	[1,2]
	1315	D Band in mesoporous carbon	[4]
	1257	SO_2 antisymmetric in $TFSI^-$	[2]
	1054	C-H deformation in PPy	[7]
	985	Pyrrole ring deformation symmetric	[8]
	929	Pyrrole ring deformation asymmetric	[8]

Figure S3. Raman spectroscopy peaks for the ionic EMIM-TFSI, polyaniline and polypyrrole as seen in Figure 4.

Figure S4

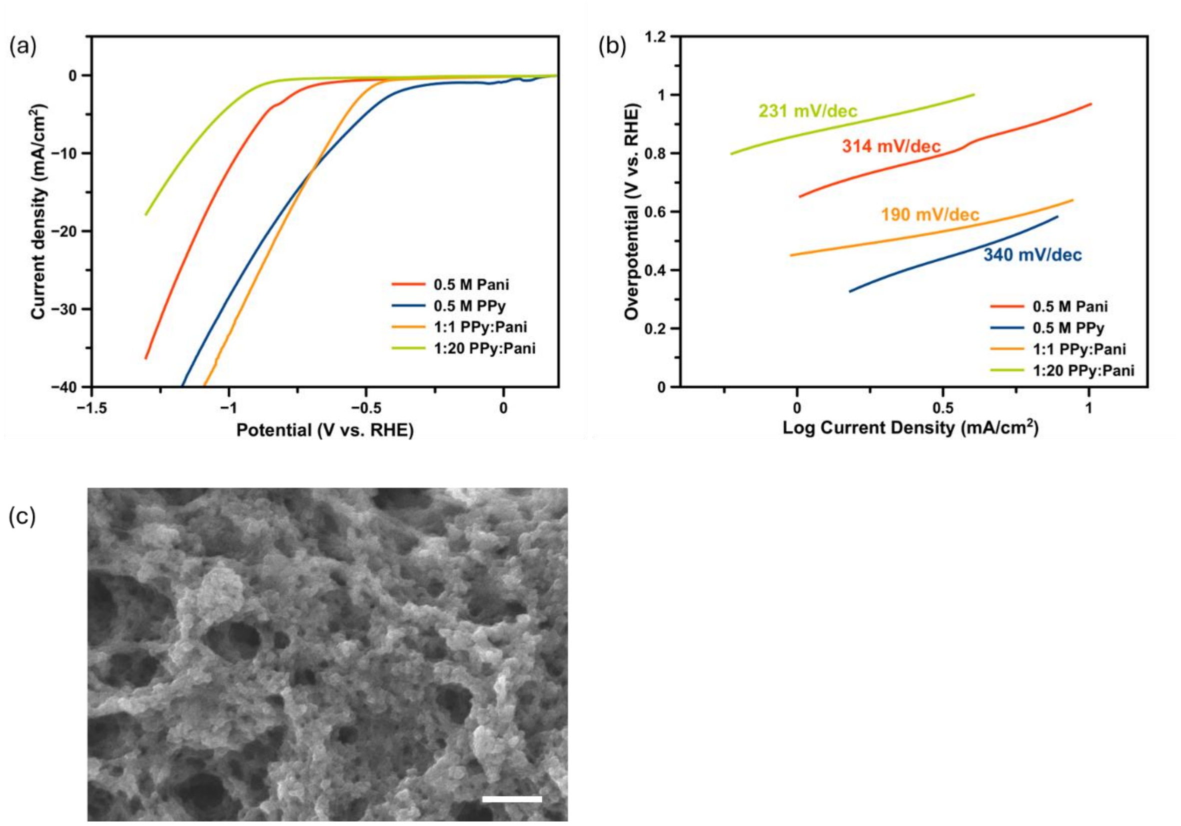

Figure S4. (a) LSV of the 1:1 and 1:20 polypyrrole: polyaniline composite with the single polymers for comparison, (b) Corresponding Tafel analysis of the 1:1 and 1:20 ratio composite again with the single polymers for comparison, (c) SEM image of the 1:1 composite showing a reduced porosity. Scale bar in image is 1 μm.

Figure S5.

(a)

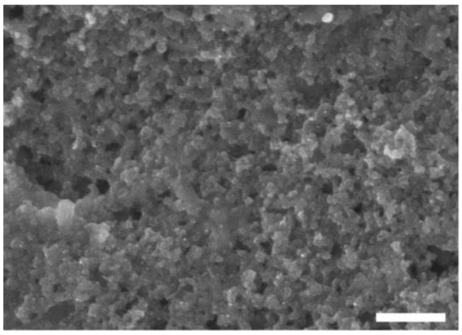

(b)

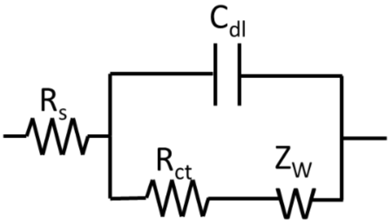

(c)

Circuit Element	Fresh 1:10 PPy: Pani	After 10,000 cycles 1:10 PPy: Pani
R_s (Ω)	112	107
C_{dl} (μF)	110	113
R_{ct} (Ω)	4.29	10.2
Z_w ($\mu\Omega$ $s^{-1/2}$)	59.4	186

(d)

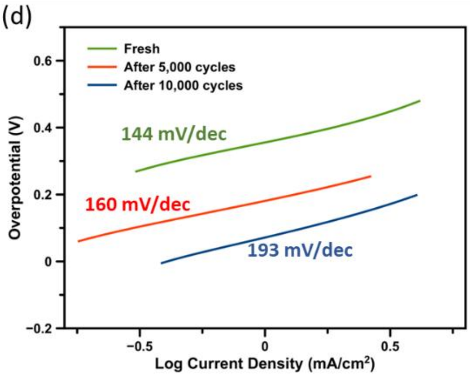

Figure S4. (a) FE-SEM image of the 1:10 polypyrrole: polyaniline film after 10,000 cycles for the stability test. Scale bar is 1 μm (b) Randles circuit used for fitting the impedance spectroscopy data (c) values for the circuit elements in the impedance spectroscopy fitting for the Randles circuit (d) Tafel analysis of the 1:10 polypyrrole: polyaniline films after stability tests.

References

[1] K. Fujii, T. Fujimori, T. Takamuku, R. Kanzaki, Y. Umebayashi, S.I. Ishiguro, *Journal of Physical Chemistry B*, **2006**, *110*, 16, 8179.

[2] A. Martinelli, A. Matic, P. Johansson, P. Jacobsson, P. Jacobsson, L. Borjesson, A. Fernicola, S. Panero, B. Scrosati, H. Ohno, *Journal of Raman Spectroscopy*, **2011**, *42*, 522.

[3] P. Sedlak, D. Sobola, A. Gajdos, R. Dallev, A. Nebojsa, P. Kubersky, *Polymers*, **2021**, *13*, 2678.

[4] J. D. Wilcox, M. M. Doeff, M. Marcinek, R. Kostecki, *Journal of the Electrochemical Society*, **2007**, *154*, 5, A389.

[5] R. Mazeikiene, G. Niaura, A. Malinauskas, *Journal of Solid State Electrochemistry*, **2019**, *23*, 1631.

[6] M. M. Nobrega, C. H. B. Silva, V. R. L. Constantino, M. L. A. Temperini, *Journal of Physical Chemistry B*, **2012**, *116*, 14191.

[7] F. Chen, G. Shi, M. Fu, L. Qu, X. Hong, *Synthetic Metals*, **2003**, *132*, 125.

[8] M. Raicopol, C. Andronescu, R. Atasiei, A. Hanganu, A. M. Manea, I. Rau, F. Kajzar, L. Pilan, *Synthetic Metals*, **2015**, *206*, 84.
